# Self‐reported quality of life in symptomatic and asymptomatic women with X‐linked adrenoleukodystrophy

**DOI:** 10.1002/brb3.2878

**Published:** 2023-02-07

**Authors:** Lisa Schäfer, Hannes Roicke, Christa‐Caroline Bergner, Wolfgang Köhler

**Affiliations:** ^1^ Department of Neurology Leukodystrophy Outpatient Clinic Leipzig University Medical Center Leipzig Germany

**Keywords:** adrenoleukodystrophy (X‐ALD), heterozygote, myeloneuropathy, quality of life, women

## Abstract

**Background:**

Up to 80% of women with X‐linked adrenoleukodystrophy (X‐ALD) develop symptoms of myelopathy and peripheral neuropathy during their lifetime. The study's objective was to compare symptomatic versus asymptomatic women with X‐ALD regarding their physical and mental well‐being and quality of life.

**Methods:**

Data were obtained from a prospective, international, cross‐sectional cohort study of women with X‐ALD recruited both clinically and population based. Symptoms, quality of life, and physical and mental co‐morbidities were assessed by questionnaires. Women were considered symptomatic if they reported any sign of myelopathy or peripheral neuropathy. Group differences between symptomatic versus asymptomatic women and between age groups were examined using *χ* ^2^ tests for categorical and independent sample *t* tests or analysis of variance for continuous variables.

**Results:**

Complete data were available from *N* = 180 women (mean age: 51.2 ± 13.6 years, range: 18–85), of whom 71.7% were classified as symptomatic, with prevalence increasing with age. Symptomatic versus asymptomatic women reported poorer physical and mental health, with 26.4% meeting the criteria for a clinical depression, 73.6% reporting chronic pain, 80.6% sleeping disturbances, 38.2% sexual dysfunction, and 47.3% restless legs syndrome. Large group differences were found on the physical health, but not on the mental health component of quality of life, where symptomatic women only differed when controlling for having a boy affected by X‐ALD (small effect) and treatment frequency (medium effect).

**Conclusions:**

Symptomatic women with X‐ALD present with physical and psychological co‐morbidities significantly reducing individuals’ quality of life. The findings emphasize the need to develop new multi‐disciplinary treatment options tailored to women's specific needs.

## Graphical abstract

This currently largest cross‐sectional cohort study of women with X‐linked adrenoleukodystrophy (X‐ALD) found that symptomatic women with X‐ALD report physical and psychological comorbidities that significantly impact their quality of life. The findings highlight the need to develop new multi‐disciplinary treatment options tailored to the specific needs of symptomatic women with X‐ALD.

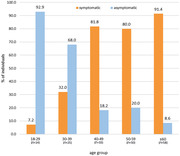

## 1 INTRODUCTION

X‐linked adrenoleukodystrophy (X‐ALD) is one of the most common inborn errors of metabolism caused by mutations in the *ABCD1* gene. The ABCD1 transporter, located at the peroxisomal membrane, shuttles activated very long chain fatty acids (VLCFA) into the organelle for further degradation. Dysfunction of the transporter in the diseases leads to toxic accumulation of saturated VLCFA in all body fluids and tissues. In males, the clinical spectrum ranges from adrenal insufficiency without neurological symptoms to a rapidly progressive, fatal cerebral demyelinating disease that usually occurs in childhood (childhood cerebral ALD [CCALD]) or adulthood (adult cerebral ALD [ACALD]) (Kemp et al., 2016; Köhler et al., 2018). The most frequent X‐ALD phenotype affecting both adult males and females is adrenomyeloneuropathy (AMN), a slowly progressive myelopathy and peripheral neuropathy, characterized primarily by progressive spastic paraparesis, sensory ataxia, and bladder and bowel dysfunction (Köhler et al., 2018; Lynch et al., 2019; Turk et al., 2020; Zhu et al., 2020), while cognitive functions generally remain unimpaired (Schäfer et al., 2021).

Due to the X‐linked inheritance, it has long been assumed that women with X‐ALD are merely carriers and remain asymptomatic. However, recent findings suggest that up to 80% of women with X‐ALD develop symptoms of myelopathy and peripheral neuropathy (Engelen et al., 2014), with gait disorders found in 65% (Engelen et al., 2014), bladder and bowel dysfunction in over 79% (Corre et al., 2021; Hofereiter et al., 2015), sensory complaints in 81% (Engelen et al., 2014), restless leg syndrome (RLS) in 48% (Winkelman et al., 2022), and neuropathic pain in over 80% of symptomatic women (Bachiocco et al., 2021; Huffnagel et al., 2019). As symptomatic courses in women progress slower, have a later onset in life (> 40 years of age), and do not usually convert to ACALD (<1%) (Engelen et al., 2014; Habekost et al., 2015; Huffnagel et al., 2019; Kemp et al., 2016; Schirinzi et al., 2019), symptoms in women with X‐ALD are frequently underestimated, overlooked, or misinterpreted, for example, as multiple sclerosis (Di Filippo et al., 2011; Stöckler et al., 1993). In addition, women with X‐ALD appear to be at higher risk for psychological distress (Kuratsubo et al., 2008), for example, depressive grief over the loss of a boy affected by CCALD and/or feeling guilty about passing the gene defect to male offspring. Further, symptomatic women with X‐ALD face particular barriers to accessing appropriate treatment on a regular basis, for example, because they are fully engaged in taking care of a diseased child or because they are not taken seriously by their local physicians who might be not aware of symptoms occurring in women with X‐ALD. Due to the slower clinical progression of symptoms in women, which is hardly detectable with current outcome measures (Huffnagel et al., 2019), women are not suitable participants in clinical trials evaluating new symptomatic treatment approaches, and curative therapy is lacking for both males and females with X‐ALD. As a consequence, many symptomatic women with X‐ALD, though often living with untreated chronic pain, bladder and bowel dysfunction, walking difficulties, depression, and psychological distress, fall out of diagnostic and therapeutic focus.

To date, research on the natural history of symptoms in women with X‐ALD and its impact on quality of life is scarce (Corre et al., 2021; Engelen et al., 2014; Hofereiter et al., 2015; Huffnagel et al., 2019). By using the well‐established Short Form (36) Health Survey (SF‐36) (Ware, 2000), two studies indicated lower quality of life related to physical disability in symptomatic versus asymptomatic women with X‐ALD, whereas no effect of symptomatic status was found on the SF‐36 mental health component (Engelen et al., 2014, Huffnagel et al., 2019). Lack of group differences in mental well‐being and its impact on quality of life may be due to relatively small sample sizes (*N* = 46–65).

Raising awareness for the needs of affected women and a deeper understanding of disease progression in women with X‐ALD appears to be crucial, finally resulting in an important contribution to the development of future treatment tailored to women's specific needs. Against this background, our study's objectives were to evaluate the impact of symptomatic courses in women with X‐ALD on various quality‐of‐life, physical, and mental health measures, and determine the additional effect of being the mother of an affected boy as well as treatment frequency on the quality of life of affected individuals in a prospective, large‐sized, both clinical and population‐based international sample.

## 2 MATERIALS AND METHODS

### 2.1 Participants and study design

Women with X‐ALD were recruited from the Leukodystrophy Outpatient Clinic of the University Hospital Leipzig, Germany, in cooperation with the European Leukodystrophy Association (ELA) International and population‐based strategies, for example, advertisement in social media and outreach to all female relatives with X‐ALD from outpatients. Eligible participants were German‐, English‐, or French‐speaking women aged ≥18 years with genetically confirmed or obligate X‐ALD based on family screening. Study participation included completion of self‐report questionnaires asking for symptoms of myelopathy and peripheral neuropathy, participants’ quality of life, and physical and mental co‐morbidities. The questionnaires were provided to participants either by mail or online via the web platform *Leuconnect* operated by ELA International (www.leuconnect.com). Informed consent was obtained from all participants in accordance with the Declaration of Helsinki. All participants were informed that analyses were conducted in a pseudonymous manner. The study protocol was approved by the Ethical Committee at the Medical Faculty, Leipzig University (473/19‐ek) and is registered on ClinicalTrials.gov (NCT04675749).

### 2.2 Neurological symptoms

Based on the disease‐specific Adult ALD Clinical Score (AACS) (Köhler & Sokolowski, 1999), a 10‐item questionnaire was developed to assess the presence of symptoms of myelopathy and/or peripheral neuropathy in women with X‐ALD on three domains: gait disorders, bladder and bowel dysfunction, and sensory complaints. Women were considered symptomatic if they reported signs of myelopathy and/or peripheral neuropathy in at least one domain.

### 2.3 Quality of life and co‐morbidities

The Short Form (36) Health Survey (SF‐36) (Ware, 2000) was used to evaluate women's health‐related quality of life on eight subscales which can be summarized into two health components: physical functioning, role‐physical (i.e., role limitations due to physical problems), bodily pain, general health (physical component), and vitality, social functioning, role‐emotional (i.e., role limitations due to emotional problems), and mental health (mental component). The eight subscales range from 0.0 (lowest quality of life) to 100.0 (highest quality of life). Total scores of the two health components were transformed to standardized *T* values (mean = 50 ± 10) according to age‐ and sex‐based norms, with higher *T* values indicating better health status.

Depressive disorders were assessed by the total score of the Beck Depression Inventory (BDI‐II, range: 0–63) (Beck et al., 1996) and participants were assigned to the following categories: 0–8 (no depression), 9–19 (mild depression), 20–28 (moderate depression), and 29–63 (severe depression).

Suicidal thinking was rated by the total score of the Beck Scale for Suicide Ideation (BSSI) (Beck & Steer, 1991), ranging from 0 to 38, with higher scores indicating stronger suicidal ideation. Participants were classified as *suicidal* if they scored >0 on item 4 (desire to make active suicide attempt) or item 5 (passive suicide desire) in the screening part of the BSSI.

Chronic pain and its impact on participants’ daily functioning was assessed by computing the total scores of the subscales pain severity (range: 0–40) and pain interference (range: 0–70) of the Brief Pain Inventory—short form (BPI‐sf) (Cleeland & Ryan, 1994) in patients who self‐reported chronic pain (item 1 of the BPI‐sf), with higher scores indicating greater pain severity and pain interference, respectively.

The total score of the Modified Fatigue Impact Scale (MFIS, range: 0–84) (Larson, 2013) was applied to measure the impact of fatigue on participants’ quality of life, with higher scores indicating greater fatigue.

Sleep quality and disturbances were assessed by the total score of the Pittsburgh Sleep Quality Index (PSQI) (Buysse et al., 1989), ranging from 0 to 21, with higher scores indicating poorer sleep. Participants were classified as *bad sleeper* if their total score was ≥5.

The total score of the Female Sexual Function Index (FSFI) (Rosen et al., 2000) was used to assess self‐reported sexual function among participating women, ranging from 4 to 95, with lower scores indicating higher sexual dysfunction. Women were classified as at risk for *sexual dysfunction* if their total score was ≤26 (Wiegel et al., 2005).

The total score of the International Restless Legs Syndrome Study Group rating scale (IRLS) (Walters et al., 2003) was used to assess the presence and severity of RLS on a range of 0 (no RLS) to 40 (very severe RLS). Based on total scores, participants were additionally assigned to the following categories: 0 (no RLS), 1–10 (mild RLS), 11–20 (moderate RLS), 21–30 (severe RLS), and 31–40 (very severe RLS).

### 2.4 Sociodemographic and control variables

Sociodemographic data included participants’ age, nationality, employment status, and treatment frequency (i.e., frequency of regular medical check‐ups for X‐ALD), which was classified *as at least every 1–2 years*, *less frequently than every 2 years*, and *no treatment at all*. To control for the impact of being a mother of an affected boy with X‐ALD on participants’ quality of life and mental and physical well‐being, women were categorized as having *no affected boy* or *at least one affected boy* for additional analyses.

### 2.5 Statistical analysis

Data were analyzed with SPSS Version 27.0, and all statistical tests were two‐tailed with a significance level set at *α* = .05. Differences between age groups and symptomatic vs. asymptomatic women regarding sociodemographic data, self‐reported symptoms, quality of life, and mental and physical co‐morbidities were examined using *χ* ^2^ tests for categorical and independent sample *t* tests for continuous variables. Analyses of variance were performed when more than two groups were compared for continuous dependent variables, and post‐hoc tests with Bonferroni correction were used to reveal the location of pair‐wise differences when omnibus tests were significant. In terms of violation of normality and homogeneity of variances, non‐parametric tests were used and reported if results differed from the parametric tests.

## 3 RESULTS

### 3.1 Sample description

The present study included data from 180 women with X‐ALD (mean age: 51.2 ± 13.6 years, range: 18−85) who were recruited internationally from November 2019 to December 2021. Most participants were from Germany (*n* = 120, 66.7%), followed by France (*n* = 30, 16.7%), the United States (*n* = 14, 7.8%), and the United Kingdom (*n* = 8, 4.4%). Based on self‐report, 129 women (71.7%) of the current sample were considered symptomatic. Symptomatic women had a higher mean age (*p* < .001), retirement rate (*p* < .001), and treatment frequency (*p* = .010) compared to asymptomatic women (Table 1).

**TABLE 1:** Sample characteristics of symptomatic and asymptomatic women with X‐linked adrenoleukodystrophy (X‐ALD)

	Symptomatic women (*n* = 129)	Asymptomatic women (*n* = 51)			
	*M* (SD, range)	*M* (SD, range)	*t*	*df*	*d*
Age (years)	55.5 (10.0, 29–85)	40.3 (15.3, 18–80)	6.559***	1, 67.7	1.30
	*n* (%)	*n* (%)	*χ* ^2^	*df*	*V*
Employment status					
Full/part time job, housewife, in training/retraining	63 (49.2)	48 (94.1)	31.753***	3, *N* = 179	0.42
Unemployment	8 (6.3)	0 (0.0)			
Disability pension	23 (18.0)	0 (0.0)			
Retirement	34 (26.5)	3 (5.9)			
Treatment frequency^a^					
At least every 1–2 years	50 (69.4)	8 (34.8)	9.279*	2, *N* = 95	0.31
Less frequently than every 2 years	11 (15.3)	6 (26.1)			
No treatment at all	11 (15.3)	9 (39.1)			

*Notes*: Effect size Cohen's *d* and Cramer's *V* were interpreted according to Cohen (small effect: 0.20 ≤ *d* < 0.50, medium: 0.50 ≤ *d* < 0.80, large: *d* ≥ 0.80; small effect: 0.10 ≤ *V* < 0.30, medium: 0.30 ≤ *V* < 0.50, large: *V* ≥ 0.50) (Cohen, 1988).

^a^ This questionnaire was added later (October 2020), causing a reduction in sample size.

* *p* < .050

*** *p* < .001.

### 3.2 Neurological symptoms

**FIGURE 1:**
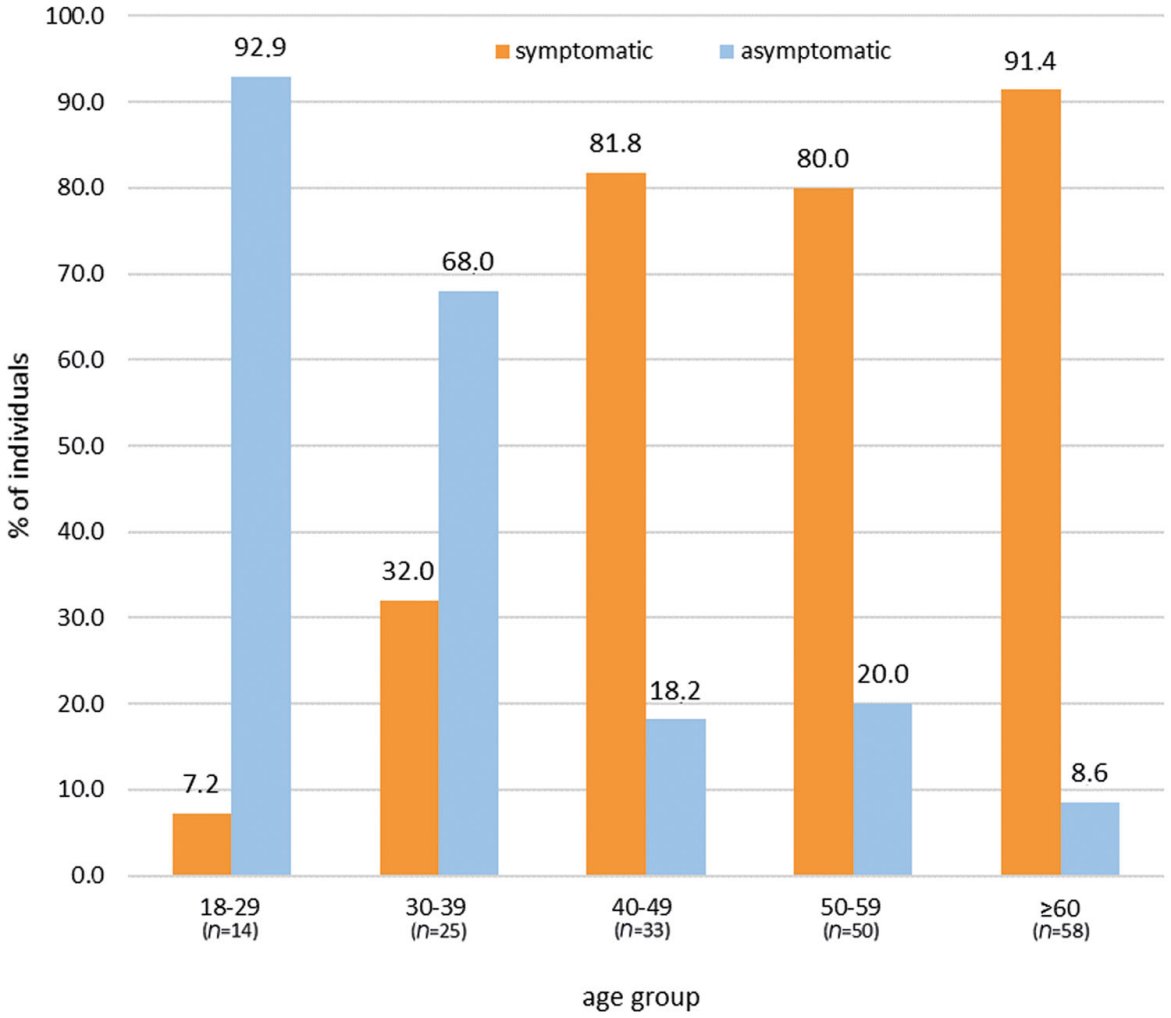
Frequency of symptomatic status by age group

Of all symptomatic women, 90.7% reported gait disorders, 80.6% bladder and bowel dysfunction, and 79.8% sensory complaints (Table 2). The prevalence of self‐reported symptoms of myelopathy and/or peripheral neuropathy steeply increased with age (*χ* ^2^ [4, *N* = 180] = 62.561, *p* < .001, *V* = 0.59; Figure 1). Severity of gait disorders increased with higher age (*p* = .036), while no age effect was found on bladder and bowel dysfunction (*p* = .795) and sensory complaints (*p* = .971).

**TABLE 2:** Description of neurological signs of myelopathy and peripheral neuropathy in symptomatic women with X‐linked adrenoleukodystrophy (X‐ALD) by age group

	18–39 Years (*n* = 9)	40–59 Years (*n* = 67)	≥60 Years (*n* = 53)	All ages (*N* = 129)		
	*n* (%)	*n* (%)	*n* (%)	*n* (%)	*χ* ^2^(2, *N* = 129)	*V*
Gait disorders	6 (66.7)	62 (92.5)	49 (92.5)	117 (90.7)	6.623*	0.23
Stiffness/pain in the legs, insecurity in walking, occasional stumbling	6 (66.7)	62 (92.5)	46 (86.8)	114 (88.4)	5.386	0.20
Walking insecurity/weakness of the legs, problems during running, sports no longer possible as before	3 (33.3)	50 (74.6)	46 (86.8)	99 (76.7)	12.670**	0.31
Walking without aids no longer possible	1 (11.1)	17 (25.4)	34 (64.1)	52 (40.3)	21.922***	0.41
Mainly movement in the wheelchair	0 (0.0)	4 (6.0)	5 (9.4)	9 (7.0)	1.273	0.10
Bladder and bowel dysfunction	8 (88.9)	54 (80.6)	42 (79.2)	104 (80.6)	0.458	0.06
Urinary hesitancy or urgency	7 (77.8)	48 (71.6)	38 (71.7)	93 (72.1)	0.155	0.04
Occasional incontinence	6 (66.7)	37 (55.2)	33 (62.3)	76 (58.9)	0.846	0.08
Permanent incontinence	2 (22.2)	17 (25.4)	10 (18.9)	29 (22.5)	0.719	0.08
Sensory complaints	7 (77.8)	54 (80.6)	42 (79.2)	103 (79.8)	0.059	0.02
Occasional loss of sensation or pain in the legs	7 (77.8)	51 (76.1)	40 (75.5)	98 (76.0)	0.024	0.01
Permanent loss of sensation or pain in the legs	3 (33.3)	29 (43.3)	29 (54.7)	61 (47.3)	2.308	0.13
Legs are completely numb	0 (0.0)	4 (6.0)	3 (5.7)	7 (5.4)	0.561	0.07

*Notes*. Effect size Cramer's *V* was interpreted according to Cohen (small effect: 0.10 ≤ *V* < 0.30, medium: 0.30 ≤ *V* < 0.50, large: *V* ≥ 0.50) (Cohen, 1988).

* *p* < .050

** *p* < .010

*** *p* < .001.

### 3.3 Quality of life and co‐morbidities

Symptomatic women reported significantly decreased health‐related quality of life on all subscales of the SF‐36 compared to asymptomatic women (*p*s ≤ .050, medium‐to‐large sized effects except for the small effect found on mental health; Table 3). A significant large‐sized group difference was found for the physical health component, which was more impacted in symptomatic compared to asymptomatic women (*p* < .001) and below average in symptomatic women compared to the norm (*T* < 40). No significant group difference emerged on the mental health component (*p* = .314). Regarding depressive disorders, symptomatic vs. asymptomatic women reported significantly higher BDI‐II total scores (*p* < .001, large effect) and showed a higher prevalence of total scores indicating a clinically relevant depression (*p* = .003, small effect). No group effect emerged on suicidal ideation assessed by BSSI (*p* = .102). Chronic pain assessed via BPI‐sf was reported more frequently by symptomatic women (*p* < .001, medium effect) and was worse in terms of pain severity (*p* = .017, medium effect) and pain interference (*p* < .001, large effect) than in asymptomatic women reporting chronic pain. Regarding fatigue, a large‐sized group effect was revealed when comparing symptomatic with asymptomatic women regarding their MFIS total scores, with higher fatigue scores in symptomatic women (*p* < .001). Sleep quality, assessed by total scores of the PSQI, was significantly decreased in symptomatic compared to asymptomatic women (*p* < .001, medium effect). Further, significantly more symptomatic women exceeded the PSQI cutoff indicating bad sleepers (*p* = .001, small effect). The groups significantly differed in self‐reported sexual function, with symptomatic women achieving lower FSFI total scores (*p* < .001, medium effect) and being more often classified as at risk for sexual dysfunction (*p* = .007, small effect) than asymptomatic women. Regarding RLS, symptomatic women scored higher in the IRLS compared to asymptomatic women (*p* < .001, medium effect), but no group differences emerged when comparing the frequency of RLS severity categories (*p* = .062, medium effect).

**TABLE 3:** Group comparison of symptomatic versus asymptomatic women with X‐linked adrenoleukodystrophy (X‐ALD) on quality of life and co‐morbidities

	Symptomatic women (*n* = 129)	Asymptomatic women (*n* = 51)			
	*M* (SD)	*M* (SD)	*t*	*df*	*d*
Quality of life (SF‐36)					
Physical functioning	47.2 (31.1)	96.2 (6.3)	−16.934***	1, 150.5	1.84
Role‐physical	40.6 (41.7)	94.0 (17.2)	−12.084***	1, 174.6	1.46
Bodily pain	51.8 (26.3)	86.7 (19.7)	−9.658***	1, 121.8	1.42
General health	46.6 (19.9)	72.2 (20.9)	−7.662***	1, 177	1.27
Vitality	42.9 (19.5)	57.5 (19.0)	−4.578***	1, 178	0.75
Social functioning	64.4 (26.7)	87.5 (19.0)	−6.491***	1, 127.7	0.93
Role‐emotional	62.2 (42.5)	85.3 (26.2)	−4.374***	1, 143.2	0.60
Mental health	61.2 (20.6)	70.0 (17.4)	−2.673**	1, 178	0.45
Physical health component	35.3 (11.9)	55.3 (5.9)	−14.815***	1, 165.4	1.89
Mental health component	45.7 (12.8)	47.5 (9.5)	−1.011	1, 120.7	0.15
Depression (BDI‐II)	14.5 (10.1)	8.4 (6.9)	4.595***	1, 129.2	−0.65
No depression, *n* (%)	39 (30.2)	29 (58.0)	*Χ* ^2^ (3, *N* = 179) = 13.942**		*V* = 0.28
Mild depression, *n* (%)	56 (43.4)	17 (34.0)			
Moderate depression, *n* (%)	21 (16.3)	3 (6.0)			
Severe depression, *n* (%)	13 (10.1)	1 (2.0)			
Suicidal ideation (BSSI)	1.2 (3.2)	0.5 (1.9)	1.673	1, 150.675	−0.22
No suicidality *n*, (%)	110 (85.3)	47 (92.2)	*Χ* ^2^ (1, *N* = 180) = 1.555		*V* = 0.09
Suicidal, *n* (%)	19 (14.7)	4 (7.8)			
Chronic pain (BPI‐sf)					
Pain severity	16.3 (7.2)	11.2 (8.5)	2.416*	1, 107	−0.69
Pain interference	28.4 (16.3)	9.3 (13.1)	4.930***	1, 19.556	−1.20
No chronic pain, *n* (%)	34 (26.4)	36 (72.0)	*Χ* ^2^ (1, *N* = 179) = 31.524***		*V* = 0.42
Chronic pain, *n* (%)	95 (73.6)	14 (28.0)			
Fatigue (MFIS)	37.1 (17.3)	17.9 (14.9)	6.960***	1, 177	−1.15
Sleep quality (PSQI)	8.2 (4.0)	5.5 (3.4)	4.255***	1, 178	−0.70
Good sleeper, *n* (%)	25 (19.4)	22 (43.1)	*Χ* ^2^ (1, *N* = 180) = 10.693**		*V* = 0.24
Bad sleeper, *n* (%)	104 (80.6)	29 (56.9)			
Sexual function (FSFI)	43.8 (31.0)	66.4 (28.0)	−4.587***	1, 94.519	0.74
No dysfunction, *n* (%)	76 (61.8)	40 (83.3)	*Χ* ^2^ (1, *N* = 171) = 7.345**		*V* = 0.21
Sexual dysfunction, *n* (%)	47 (38.2)	8 (16.7)			
Restless legs syndrome (IRLS)^a^	10.3 (11.2)	2.5 (5.0)	4.405***	1, 72.414	−0.77
No RLS, *n* (%)	39 (52.7)	19 (79.2)	*Χ* ^2^ (4, *N* = 98) = 9.230		*V* = 0.31
Mild RLS, *n* (%)	5 (6.7)	3 (12.5)			
Moderate RLS, *n* (%)	15 (20.3)	2 (8.3)			
Severe RLS, *n* (%)	10 (13.5)	0 (0.0)			
Very severe RLS, *n* (%)	5 (6.8)	0 (0.0)			

*Notes*: Effect sizes Cohen's *d* and Cramer's *V* were interpreted according to Cohen (small effect: 0.20 ≤ *d* < 0.50, medium: 0.50 ≤ *d* < 0.80, large: *d* ≥ 0.80; small effect: 0.10 ≤ *V* < 0.30, medium: 0.30 ≤ *V* < 0.50, large: *V* ≥ 0.50) (Cohen, 1988). SF‐36 = Short Form (36) Health Survey (subscales: 0*−100, less favorable scores are asterisked; health components: *T* values according to age‐ and sex‐based norms); BDI‐II = Beck Depression Inventory (0–63*); BSSI = Beck Scale for Suicide Ideation (0–38*); BPI‐sf = Brief Pain Inventory – short form (severity: 0–40*; interference: 0–70*); MFIS = Modified Fatigue Impact Scale (0–84*); Pittsburgh Sleep Quality Index (0–21*); FSFI = Female Sexual Function Index (4*−95); IRLS = International Restless Legs Syndrome Study Group rating scale (0–40*); RLS = Restless Legs Syndrome.

^a^ This questionnaire was added later (October 2020), causing a reduction in sample size.

* *p* < .050

** *p* < .010

*** *p* < .001.

### 3.4 Effect of control variables

Among the group of symptomatic women, mothers of at least one affected boy with X‐ALD significantly differed from symptomatic women with no affected boy on the SF‐36 subscale social functioning (*M*_+boy_ = 60.1 ± 25.6 vs. *M*_‐boy_ = 72.2 ± 26.5; *t*[1, 126] = −2.553, *p* = .012, *d* = −0.46) and the SF‐36 mental health component (*M*_+boy_ = 44.0 ± 12.3 vs. *M*_‐boy_ = 49.1 ± 13.0; *t*[1, 123] = −2.205, *p* = .029, *d* = −0.41), with symptomatic mothers of boys with X‐ALD reporting greater impairment. Asymptomatic women with versus without at least one affected boy significantly showed higher impairment on the SF‐36 subscale bodily pain (*M*_+boy_ = 77.5 ± 22.9 vs. *M*_‐boy_ = 90.7 ± 16.6; *t*[1, 48] = −2.376, *p* = .022, *d* = −0.71). No further effects were detected in the symptomatic or asymptomatic group (*p*s > .050).

Controlling for treatment, symptomatic women with regular medical check‐ups for X‐ALD at least every 1–2 years scored significantly lower on the SF‐36 subscale physical functioning (*M*_<2years_ = 41.2 ± 28.5 vs. *M*_>2years_ = 68.6 ± 25.3 vs. *M*_noTreat_ = 63.8 ± 23.0; *F*[2, 69] = 7.479, *p* = .001, *η* ^2^ = 0.18), physical role (*M*_<2years_ = 28.5 ± 37.8 vs. *M*_>2years_ = 59.1 ± 43.7 vs. *M*_noTreat_ = 61.4 ± 43.8; *F*[2, 69] = 4.908, *p* = .010, *η* ^2^ = 0.12), and on the SF‐36 physical health component (*M*_<2years_ = 32.1 ± 10.8 vs. *M*_>2years_ = 43.0 ± 10.2 vs. *M*_noTreat_ = 44.0 ± 12.8; *F*[2, 69] = 8.231, *p* < .001, *η* ^2^ = 0.19) compared to symptomatic women having check‐ups less frequently or not at all. Further, symptomatic women with check‐ups every 1–2 years showed significantly lower impairment on the SF‐36 subscale mental health (*M*_<2years_ = 60.4 ± 20.0 vs. *M*_>2years_ = 53.5 ± 18.7 vs. *M*_noTreat_ = 43.6 ± 16.8; *F*[2, 69] = 3.563, *p* = .034, *η* ^2^ = 0.09) and on the SF‐36 mental health component (*M*_<2years_ = 45.2 ± 12.2 vs. *M*_>2years_ = 41.4 ± 14.8 vs. *M*_noTreat_ = 34.6 ± 10.7; *F*[2, 69] = 3.365, *p* = .040, *η* ^2^ = 0.09), and reported lower depression scores (BDI‐II) compared to symptomatic women seeking no treatment at all (*M*_<2years_ = 15.2 ± 9.3 vs. *M*_>2years_ = 14.9 ± 10.7 vs. *M*_noTreat_ = 23.7 ± 13.3; *F*[2, 69] = 3.316, *p* = .042, *η* ^2^ = 0.09), while no other significant subgroup differences emerged (*ps* > .050). No further significant differences were detected in the symptomatic or asymptomatic group regarding treatment frequency (*p*s > .050).

## 4 DISCUSSION

To date, physical and mental well‐being and its impact on quality of life in symptomatic vs. asymptomatic women with X‐ALD have never been studied in detail, for example, using eligible self‐report questionnaires. This currently largest, prospective, cross‐sectional cohort study of women with X‐ALD found that the quality of life in symptomatic women is tremendously impaired in several areas of daily life and well‐being, with 43.4% of the present sample meeting the criteria for a mild and 26.4% for a moderate to severe depression requiring treatment, 14.7% reporting suicidal ideation, 73.6% chronic pain, 80.6% sleeping disturbances, 38.2% sexual dysfunction, and 47.3% RLS. Thus, the physical and psychological distress of symptomatic women significantly exceeds that found in the general population, where rates of 7.7%–15.0% for clinical depression (Arias‐De la Torre et al., 2021; McGee & Thompson, 2010), 4.0%–7.9% for suicidal ideation (Forkmann et al., 2012; Ivey‐Stephenson et al., 2022; Kliem et al., 2017; Legleye et al., 2010), 20.4%–32.7% for chronic pain (Chenaf et al., 2018; Ohayon & Stingl, 2012; Zelaya et al., 2020), 31.0%–56.0% for sleeping disturbances (Hinz et al., 2017; Léger et al., 2008), 17.5%–43.0% for sexual dysfunction (Briken et al., 2020; Laumann et al., 1999; McCool et al., 2016), and 3.9%–14.3% for RLS (Allen et al., 2005; Ohayon et al., 2012) have been reported. Further, symptomatic women reported high fatigue scores (*M_ALD_* = 37.1 ± 17.3) comparable to those of patients with multiple sclerosis (*M_MS_* = 41.1 ± 17.1) and exceeding those of healthy individuals (*M_NORM_* = 15.3 ± 12.0) (Strober et al., 2020). The results of the present study highlight the need for a multi‐approach diagnostic and treatment process in symptomatic women with X‐ALD that not only focuses on disease‐specific symptoms such as spasticity and bladder and bowel dysfunction, but takes into account multiple physical and mental co‐morbidities that affect the individual's quality of life.

Although determined by self‐report, the prevalence of RLS found in the present sample of symptomatic women with X‐ALD (47.3%) is consistent with recent findings based on diagnostic telephone interviews (47.6%) (Winkelman et al., 2022). Of note, a significant proportion of women (20.8%) who were classified as asymptomatic due to the absence of signs of myelopathy and peripheral neuropathy reported mild to moderate symptoms of RLS, which were above the prevalence rate found in the general population (3.9%–14.3%) (Allen et al., 2005; Ohayon et al., 2012), while the prevalence rates of other physical and mental co‐morbidities were within the normal range. This finding suggests that RLS in asymptomatic women with X‐ALD might be an early sign of myelopathy; however, this question should be investigated in future studies with longitudinal designs.

In contrast to previous findings (Engelen et al., 2014; Huffnagel et al., 2019), symptomatic vs. asymptomatic women differed significantly on all eight SF‐36 subscales assessing quality of life, with large effects found on subscales measuring quality of life related to physical disability (*d* = 1.27–1.84) and small‐to‐medium effects on subscales assessing quality of life related to mental health (*d* = 0.45–0.93). Consistent with (Huffnagel et al. (2019), symptomatic vs. asymptomatic women differed significantly on the physical health component (large effect), with symptomatic women exhibiting levels far below the norm (T = 35.3), while no group differences were found on the SF‐36 mental health component, in which both groups scored within the norm range (*T* > 40). However, sub‐analyses revealed that symptomatic mothers of at least one boy affected by X‐ALD significantly differed from symptomatic women without a diseased boy on the SF‐36 subscale social functioning and the mental health component, indicating that being a parent and caregiver of a diseased child rather than one's own physical disability seems to have a greater impact on mental health and social factors. Indeed, previous research suggests poor correlation between physical and mental health status in symptomatic women with X‐ALD (Engelen et al., 2014) but impaired psychological and social functioning in parents of children with a rare disease (Boettcher et al., 2021; Kuratsubo et al., 2008; Pelentsov et al., 2016). This again highlights the importance of a multi‐disciplinary approach in the clinical care of women with X‐ALD, which, in addition to symptomatic treatment of neurological symptoms, also takes into account psychological support and social work (Dimitrova et al., 2021), especially for women with familial clustering of the disease and particular burden of caring for relatives.

Consistent with previous, mainly clinically recruited cohort studies, the prevalence of self‐reported symptoms in women increased steeply with age, finally affecting more than 91% of women older than 60 years. The current data support the previously reported cut‐off of 41 years being crucial for symptom onset in more than 80% of women with X‐ALD (Schirinzi et al., 2019). In addition, symptoms were already present in approximately 20% of women younger than 40 years in the current sample (Azar et al., 2020), and a few women were asymptomatic in self‐report even in old age. However, the prevalence rates for symptomatic courses in women with X‐ALD found in our and previous samples should be interpreted with caution and most likely represent an overestimation of the actual prevalence in the general population due to recruitment bias. Despite great efforts to also reach women with X‐ALD from the general population and across age range, 46.1% of the present sample comprised women aged 40–59 years, mainly symptomatic and clinically recruited. It remains unclear how high the real prevalence of asymptomatic women in the general population is who are unaware of their gene defect, or of symptomatic women running under a false diagnosis, for example, multiple sclerosis (Di Filippo et al., 2011; Stöckler et al., 1993), before receiving a proper diagnosis at later ages. Thus, estimating the real prevalence of symptomatic courses in women with X‐ALD remains challenging due to these biases that cannot be overcome as long as X‐ALD is not included in newborn screening in both genders (Lee et al., 2020; Matteson et al., 2021).

Whereas the presence and severity of gait disorders in symptomatic women increased by age (from 66.7% in women <40 years to 92.5% in women >60 years of age), bladder and bowel dysfunction and sensory complaints, even of high severity, were common symptoms in women independent of age (>80%). The self‐reported prevalence rates are consistent with recent findings from studies using objective measures, such as those assessing bladder and bowl dysfunction in women with X‐ALD (Corre et al., 2021). Remarkably, 66.7% of young symptomatic women reported occasional incontinence and 33.3% permanent loss of sensation or pain in the legs. This finding highlights the need to consider rare diseases like X‐ALD in the differential diagnosis of women presenting to their physician with these complaints and unclear etiology.

In terms of treatment frequency, the current data show that more symptomatic women than asymptomatic women seek medical check‐ups for X‐ALD every 1–2 years. In addition, sub‐analyses showed that high physical disability was associated with higher treatment frequency among symptomatic women, while those without X‐ALD‐specific treatment reported significantly lower mental health and higher depression scores. This may suggest that regular medical check‐ups for X‐ALD improve the mental well‐being of symptomatic women and/or that high psychological distress and depression are barriers to seeking X‐ALD‐specific treatment. Thus, 30.6% of symptomatic women in our sample do not present for regular check‐ups (<2 years), although recommended (Engelen et al., 2012), and 39.1% of asymptomatic women do not seek medical evaluation at all. This suggests that the clinical management of women with X‐ALD is still lagging behind the standards of men and that there is a high need for raising awareness and breaking through barriers.

Strengths of this study include the prospective design, the large sample size allowing for even detecting small‐to‐medium sized effects, and the international clinical‐ and population‐based recruitment. Physical and mental status and its impact on quality of life were assessed multi‐dimensionally using various state‐of‐the‐art questionnaires. The results of the present study are limited by the assessment of myelopathy and peripheral neuropathy symptoms and co‐morbidities in self‐reports, risking over‐ or underreporting. Further, the results of the present study might be biased by additional effects of the COVID‐19 pandemic on individuals’ psychological well‐being and quality of life (Chung et al., 2020; Halley et al., 2021). Moreover, due to the cross‐sectional design, no conclusions can be drawn about causal relationships.

In summary, the current study indicates a high prevalence of physical and psychological co‐morbidities in symptomatic women with X‐ALD, with significant impact on individuals’ quality of life. Physicians are advised to perform a comprehensive diagnostic assessment in women with X‐ALD and develop a therapy plan that goes beyond the relief of symptoms of myelopathy and peripheral neuropathy only. Future longitudinal clinical studies should focus on improving the quality of life of symptomatic women with X‐ALD, for example, by developing and evaluating the effectiveness of new multi‐disciplinary interventions that provide additional psychological and social support.

## AUTHOR CONTRIBUTIONS

**Christa‐Caroline Bergner**: Methodology (equal) and writing—review and editing (equal); **Hannes Roicke**: Conceptualization (supporting), methodology (equal), and writing—review and editing (equal): **Wolfgang Köhler**: Methodology (equal) and writing—review and editing (equal); **Lisa Schäfer**: Conceptualization (lead), methodology (equal), formal analysis (lead), writing—original draft (lead), and writing—review and editing (equal).

## CONFLICTS OF INTEREST

The authors declare no conflict of interest.

### PEER REVIEW

The peer review history for this article is available at https://publons.com/publon/10.1002/brb3.2878

## Data Availability

The data that support the findings of this study are available on request from the corresponding author. The data are not publicly available due to privacy or ethical restrictions.
